# Treatment effect modifiers of virtual reality-based versus standard cognitive behavioral therapy for paranoia in schizophrenia spectrum disorders: an exploratory moderator analysis of clinical and demographic characteristics in the FaceYourFears trial

**DOI:** 10.1017/S0033291726103870

**Published:** 2026-04-27

**Authors:** Mads Juul Christensen, Ditte Lammers Vernal, Ulrik Nykjær Jeppesen, Carsten Hjorthøj, Jan Mainz, Amy Pinkham, Stephen Austin, Wim Veling, Thomas Ward, Merete Nordentoft, Louise Birkedal Glenthøj

**Affiliations:** 1 Department of Psychiatry, Aalborg University Hospital, Aalborg, North Denmark Region, Denmark; 2 Department of Clinical Medicine, Aalborg University, Denmark; 3 Department of Psychology, University of Copenhagen, Denmark; 4 VIRTU Research Group, Mental Health Center Copenhagen, Copenhagen University Hospital – Mental Health Services CPH, Denmark; 5 Department of Public Health, University of Copenhagen, Denmark; 6 Copenhagen Research Centre for Mental Health (CORE), Capital Region, Denmark; 7 Department of Health Economics, University of Southern Denmark, Odense, Denmark; 8 School of Behavioral and Brain Sciences, University of Texas at Dallas, USA; 9 Mental Health Services East, Copenhagen University Hospital – Psychiatry Region Zealand, Roskilde, Denmark; 10 Centre of Psychiatry, University Medical Center Groningen, Faculty of Medical Sciences, University of Groningen, Netherlands; 11 South London & Maudsley NHS Foundation Trust, London, UK; 12 Department of Psychology, Institute of Psychiatry, Psychology & Neuroscience, King’s College London, London, UK; 13 Department of Clinical Medicine, Faculty of Health and Medical Sciences, University of Copenhagen, Denmark

**Keywords:** cognitive behavioral therapy, delusions, effect modifier, immersive virtual reality, paranoia, psychosis, schizophrenia spectrum disorders

## Abstract

**Background:**

This exploratory study examined baseline characteristics modifying treatment effects on paranoia in individuals diagnosed with schizophrenia spectrum disorders following a 10-session virtual reality-based cognitive behavioral therapy for psychosis (VR-CBTp) or standard CBTp.

**Methods:**

All participants in the FaceYourFears trial were included (*n*=254; CBTp *n*=128; VR-CBTp *n*=126). General linear and logistic regression models examined baseline variables associated with end-of-treatment paranoia. In covariate analyses, regression coefficients quantified associations across treatments. In moderation analyses, interaction terms (randomization x moderator) were tested, with corresponding regression coefficients estimated and assessed at the 25th (low), 50th (medium), and 75th (high) percentiles of continuous variables.

**Results:**

Across treatments, higher baseline avolition, safety behavior, delusion severity, and cognitive biases were associated with end-of-treatment paranoia. Moderation analyses revealed interactions for avolition, delusion severity, and negative other-beliefs. Although avolition and delusion severity were associated with poorer outcomes overall, individuals with high avolition and those with moderate-to-high delusion severity improved more in VR-CBTp than CBTp, whereas participants with lower delusion severity showed better outcomes in CBTp. No demographic (age, gender, education, and occupation) or other clinical characteristics (diagnosis, paranoia, social anxiety, depression, anhedonia, total negative symptoms, functioning, core beliefs, or interpersonal trauma) were significantly associated with outcome.

**Conclusions:**

This exploratory study generates hypotheses for future research, including VR-CBTp’s potential to engage individuals with high avolition. Given the modest effects and largely nonsignificant findings, both CBTp and VR-CBTp appear suitable for a wide range of individuals with paranoia, highlighting the importance of considering patient preferences.

## Introduction

Paranoia, or ideas of social self-reference and persecution, is predominantly perceived to exist within the general population on a continuum ranging from a nonclinical phenomenon through paranoid ideation to persecutory delusions (e.g. Freeman et al., 2025). Delusions are highly individual and complex, emerging from interactions among multiple factors, including internal psychological processes as well as social and cultural influences (Ritunnano et al., 2022). Although delusions occur transdiagnostically (Pappa et al., 2025; Rootes-Murdy et al., 2022), they are frequent, often persistent, and associated with poorer long-term recovery in schizophrenia spectrum disorders (SSDs) (Feyaerts et al., 2021; Grunfeld et al., 2024; Harrow & Jobe, 2010; Rosen et al., 2022). While recovery is possible in SSD, outcomes remain suboptimal for many individuals, highlighting the need for more effective interventions (Molstrom et al., 2022).

Pharmacological treatment can be effective in managing positive symptoms in SSD, such as delusions (Leucht et al., 2025). However, issues with adverse effects (Huhn et al., 2019), discontinuation (Brandt et al., 2022), nonadherence (Kishimoto et al., 2021), suboptimal response (O’Donoghue et al., 2024), and residual symptoms (Schennach et al., 2015) remain. Together with ongoing debate regarding long-term antipsychotic use relative to short-term benefits (Bjornestad et al., 2020; Schlier et al., 2023), this highlights the need to expand access to effective nonpharmacological interventions.

Cognitive behavioral therapy for psychosis (CBTp) is a recommended first-line psychological treatment, yet rates of receipt remain low (Burgess-Barr et al., 2023). Meta-analytic evidence from an umbrella review suggests small-to-moderate effects of CBTp on delusions (Berendsen et al., 2024). While the effectiveness seems to have improved over time (Sitko et al., 2020), CBTp neither consistently outperforms other psychological treatments (Varese et al., 2025), nor demonstrates sustained treatment effects (Berendsen et al., 2024; Mehl et al., 2015). CBTp targeting specific symptoms (e.g. delusions) may be more effective than generic approaches addressing psychosis broadly (Freeman et al., 2019; Lincoln & Peters, 2019). Emerging evidence, such as from the Feeling Safe Programme (Freeman et al., 2021), supports its benefits in treating persecutory delusions. These approaches are grounded in interventionist causal models (Kendler & Campbell, 2009), aiming to identify and modify mechanisms that maintain distressing symptoms (Freeman 2024a, 2024b).

Virtual reality-assisted CBTp (VR-CBTp) enables more targeted interventions for paranoia (Bird et al., 2024), with promising results (Freeman et al., 2016; Pot-Kolder et al., 2018). Compared to traditional therapies, VR-CBTp has the potential to be more engaging, with immersive VR eliciting cognitions, emotions, and behaviors in a safe environment, potentially increasing motivation and treatment adherence (Bell et al., 2024). In the recent FaceYourFears trial, 10 sessions of CBTp or VR-CBTp targeting paranoia in individuals with SSD produced large within-group reductions in paranoia (Jeppesen et al., 2025). However, no between-group superiority was found, mirroring results from a similar trial (van der Stouwe et al., 2025). Moreover, efficacy remains uncertain in the absence of an inactive control group. Combined with the additional implementation barriers of digital interventions like VR (Bin et al., 2025), identifying treatment effect modifiers is crucial to clarify who benefits and assess VR’s added value.

In CBTp, a systematic review suggested that female gender, older age, greater clinical insight, shorter illness duration, and higher educational attainment were associated with better outcomes (O’Keeffe et al., 2017). In contrast, higher baseline negative symptoms have been associated with poorer treatment response (e.g. Lincoln et al., 2014; Thomas et al., 2011), with avolition identified as a key factor (Allott et al., 2011; Strauss et al., 2021). Moreover, greater delusion severity may interfere with treatment response (Brabban et al., 2009), although findings remain mixed (Lincoln et al., 2014). Especially, high conviction may delay therapy response and reduce the likelihood of a positive response (Jenner et al., 2024). Relatedly, cognitive biases have been linked to delusions (De Rossi & Georgiades, 2022) and may maintain paranoia by limiting consideration of alternative interpretations (Freeman, 2016; Mehl et al., 2014). However, meta-analytic evidence indicates no consistent effect of treatment modification by demographic or clinical variables (Turner et al., 2020; Varese et al., 2025). Most studies evaluated generic CBTp rather than symptom-specific approaches, and few examined specific symptoms – such as paranoia – as variables associated with outcomes, limiting understanding of factors influencing targeted CBTp response (Thomas, 2015). In VR interventions, higher baseline safety behaviors have been associated with improved outcomes in VR-CBTp for paranoia (Berkhof et al., 2024) and in automated VR therapy for agoraphobia (Freeman et al., 2022). These findings warrant replication and further investigation, ideally through direct comparisons between CBTp and VR-CBTp.

Identifying treatment effect modifiers can inform the selection of interventions based on individual symptom profiles (Hofmann & Hayes, 2019). An important next step is therefore to determine whether CBTp and VR-CBTp share similar modifiers of paranoia outcomes or differ in meaningful ways. To guide this investigation, we propose three categories of baseline variables, namely those (1) previously examined in CBTp-studies (O’Keeffe et al., 2017; Turner et al., 2020; Varese et al., 2025); (2) involved in the formation and maintenance of paranoia (Freeman, 2016; Freeman & Loe, 2023; Freeman et al., 2021; Valmaggia, Day, & Rus-Calafell, 2016); and (3) clinically or theoretically relevant explored more tentatively, reflecting VR-CBTp’s early stage (Bell et al., 2024), particularly in domains where VR shows promise (Freeman et al., 2024, 2022; Jongeneel et al., 2018; Novo et al., 2021; Pot-Kolder et al., 2018; Schröder et al., 2023; Schroeder et al., 2022; Veling et al., 2016).

Accordingly, this exploratory study investigated whether baseline characteristics were associated with post-treatment paranoia and whether they interacted with treatment allocation among individuals with SSD following 10 sessions of CBTp or VR-CBTp in the FaceYourFears trial.

## Methods

### Study design and source of data

This exploratory moderator study used baseline data collected before randomization and outcome data at treatment cessation (3 months) from FaceYourFears, a two-site, assessor-masked, randomized, parallel-group superiority trial comparing VR-CBTp (*n* = 126) with CBTp (*n* = 128) for paranoia in SSD. Elsewhere, more details are provided on FaceYourFears’ methodology (Jeppesen et al., 2022), outcomes (Jeppesen et al., 2025), and secondary analyses of mechanisms of change (Jeppesen et al., n.d.).

### Participants

The target population was individuals diagnosed with SSD experiencing paranoia. Inclusion criteria were age ≥ 18 years, SSD diagnosis (ICD-10, F20–29), ability to provide informed consent, and scoring ≥40 on the Green Paranoid Thought Scale (GPTS) (Green et al., 2008). Exclusion criteria were organic brain disease, IQ ≤ 70, inability to tolerate assessment, or insufficient Danish proficiency (Jeppesen et al., 2022). The trial was conducted across two Danish sites. Enrolment started March 26, 2021; follow-up ended August 10, 2024.

### Sample size

The sample comprised all 270 randomized participants in the FaceYourFears trial. Because the trial was powered for its primary hypotheses rather than the secondary analyses reported here, and because power calculations are hypothesis- and dataset-specific, post hoc power calculations were not conducted.

### Interventions

The FaceYourFears trial adapted therapy manuals from previous research (Pot-Kolder et al., 2018), delivering 10 individual sessions focused on paranoia. Trained psychologists provided both treatments (Jeppesen et al., 2025).

CBTp targeted paranoia using a cognitive model emphasizing aberrant salience, reasoning biases, and consolidation processes. Sessions included cognitive worksheets, psychoeducation, cognitive restructuring, core belief work, and self-worth enhancement, with guided homework and in vivo experiments where feasible.

VR-CBTp integrated CBTp principles with in-session exposure and behavioral experiments in immersive VR using CleVR Social Worlds. Participants confronted paranoia triggers in realistic, customizable virtual settings, enabling therapists to intervene in real time during ‘hot’ cognitions.

### Candidate baseline variables

In the current study, all candidate baseline variables, whether previously tested in CBTp or newly examined in VR-CBTp, were theoretical in nature, grounded in cognitive-behavioral models of psychosis. Consistent with the conceptual framework outlined in the Introduction, variables were organized into three overarching categories: (1) established CBTp moderators, (2) variables related to mechanisms underlying paranoia, and (3) exploratory VR-CBTp-relevant variables. For conceptual coherence, the variables were organized hierarchically as follows: Established CBTp variables, theoretically relevant but untested VR variables, trauma-related variables, and supplementary psychosocial factors.

The established CBTp variables included age, gender, positive symptoms, negative symptoms, anxiety, and depression (Varese et al., 2025). These were supplemented by education, diagnosis, paranoid ideation, social anxiety, safety behavior, core beliefs, and social functioning, which had been examined in a VR-CBTp versus TAU moderator study (Berkhof et al., 2024). Variables tested in CBTp but not yet in VR-CBTp were added, including avolition and anhedonia (Allott et al., 2011), cognitive biases (Lincoln et al., 2014), and symptom severity (Allott et al., 2011; Lincoln et al., 2014; Naeem et al., 2008; Tarrier et al., 1993, 1998) conceptualized as delusion severity. Interpersonal trauma was included due to its established association with paranoia (Frost et al., 2024; Grady et al., 2024; Jorovat et al., 2025), and prior studies examining the moderation of post-traumatic stress symptoms (de Haan et al., 2024) and childhood trauma (Spidel et al., 2019) on therapy outcomes. Finally, given VR-CBTp’s early stage, supplementary analyses included psychosocial functioning (Schroeder et al., 2022), self-esteem (Jongeneel et al., 2018), personality traits (Katifori et al., 2022), social cognition (Pinkham et al., 2018), basic symptoms (Schultze-Lutter, 2009), and additional demographics to inform the field and future research (Supplementary Appendix A).

### Measures

**Paranoia ideation** was assessed using the *Revised Green Paranoid Thoughts Scale* (R-GPTS) (Freeman et al., 2021) as a candidate baseline variable, due to its strong psychometric properties, while the original GPTS (Green et al., 2008) was retained as the outcome variable to align with the primary study (Jeppesen et al., 2025).

**Safety behavior** was assessed by the *Safety Behaviour Questionnaire* (Freeman et al., 2001) with the addition of close-ended questions for in-situation behaviors in FaceYourFears (Jeppesen et al., 2025).

**Delusion severity** was assessed by the *Scale for the Assessment of Positive Symptoms* (SAPS; Andreasen, 1984) subscale global delusion severity.

**Negative symptoms** were assessed by the *Brief Negative Symptom Scale*’s (BNSS; Kirkpatrick et al., 2011; Strauss et al., 2012) total score, and the subscales anhedonia and avolition.

**Social anxiety** was assessed by the *Social Interaction Anxiety Scale* (SIAS; Mattick & Clarke, 1998).

**Depression** was assessed by the *Calgary Depression Scale for Schizophrenia* (CDS; Addington et al., 1993).

**Cognitive biases** were assessed by the *Davos Assessment of Cognitive Biases Scale* (DACOBS; Van der Gaag et al., 2013).

**Core beliefs** were assessed by the *Brief Core Schema Scale* (BCSS; Fowler et al., 2006).

**Level of function** was assessed by the *Personal and Social Performance scale* (PSP; Morosini et al., 2000).

**Interpersonal trauma** was assessed by the *Trauma and Life Events Checklist* (TALE; Carr et al., 2018) using a composite score of interpersonal trauma types.

**Education** was self-reported and categorized according to the highest level achieved: primary education, vocational education, secondary/high school education, post-secondary education, or other.

### Statistical analyses

Using Stata 18.0, separate general linear models (continuous variables) and logistic regression models (categorical variables) examined whether baseline demographic and clinical variables (Table 1) were associated with paranoia reduction at treatment cessation (GPTS total score at 3 months) in VR-CBTp and/or CBTp. Following the intention-to-treat principle, all randomized participants were included in the analyses.

**Table 1.** Baseline participant demographics and clinical characteristics

	VR-CBT *M* (SD), *n* = 126*	CBT *M* (SD), *n* = 128*	Total *M* (SD), *n* = 254
Age	30,6 (10,7)	28,8 (9,8)	29,7 (10,2)
Male (%)	42.9%	42.2%	42.50%
Level of education			
Primary	30.2%	34.4%	32.3%
Vocational	13.5%	9.4%	11.4%
Secondary/high school	34.1%	39.8%	37.0%
Post-secondary	19.8%	14.1%	16.9%
Other	2.4%	2.3%	2.4%
Clinical diagnosis (%)			
Schizophrenia (F20.0–9)	74.6%	70.3%	72.4%
Schizotypy (F21)	15.9%	21.1%	18.5%
Other (F22–9)	9.5%	8.5%	9.1%
Baseline paranoid ideation, R-GPTS	28.3 (14)	29.3 (15.2)	28.8 (14.6)
Baseline social anxiety, SIAS	46.8 (14.5), *n* = 126	47.8 (15.2), *n* = 127	47.3 (14.85)
Baseline depressive symptoms, CDSS	6.1 (4), *n* = 124	5.7 (3.6), *n* = 126	5.9 (3.8)
Baseline safety behavior, SBQ	55.1 (23,2)	54.2 (24.2)	54.7 (23.7)
Baseline positive self-beliefs, BCSS	8.1 (5,4)	7.5 (4.6)	7.8 (5.0)
Baseline negative self-beliefs, BCSS	10.1 (5,3)	10.2 (5.1)	10.2 (5.2)
Baseline positive other beliefs, BCSS	9.1 (4,4)	8.5 (3.7)	8.8 (4.1)
Baseline negative other beliefs, BCSS	7.5 (5)	8.7 (5.1)	8.1 (5.0)
Baseline level of functioning, PSP	42.7 (12.6)	45.3 (12.9)	44.0 (12.8)
Baseline delusion severity, SAPS	3.9 (0.9)	3.7 (1.0)	3.8 (1.0)
Baseline negative symptoms, BNSS	23.4 (10.0)	23.3 (11.1), *n* = 126	23.4 (10.5)
Baseline anhedonia, BNSS	2.6 (1.3)	2.5 (1.4)	2.5 (1.3)
Baseline avolition, BNSS	1.6 (1.1)	1.5 (1.1)	1.1 (1.1)
Baseline cognitive bias, DACOBS	177.0 (26.6)	177.8 (26.4)	177.4 (26.4)
Baseline interpersonal trauma, TALE	3.6 (2.3)	3.4 (2.3)	3.5 (2.3)

Note: * = unless otherwise specified. Measures: BCSS: Brief Core Schema Scale; BNSS: Brief Negative Symptom Scale; CDSS: Calgary Depression Scale for Schizophrenia; DACOBS: Davos Assessment of Cognitive Biases Scale; PSP: Personal and Social Performance Scale; R-GPTS: Revised Green Paranoid Thoughts Scale; Safety Behavior Questionnaire; SAPS: Scale for the Assessment of Positive Symptoms; SIAS: Social Interaction Anxiety Scale; TALE: Trauma and Life Events Checklist.

Analysis of covariance (ANCOVA), adjusting for baseline scores as a covariate, was used to estimate treatment effects at post-treatment. ANCOVA focusing on the end-point score was selected in accordance with recommendations for RCTs (Clifton & Clifton, 2019; Fu et al., 2015; Van Breukelen, 2006), as it accounts for baseline group differences and avoids potential bias associated with change scores, including regression to the mean. Each candidate’s baseline variable was analyzed in a separate model. The significance level was set to *p* < 0.05 with a two-sided 95% confidence interval (CI). Post hoc analyses were conducted to examine subscale scores for measures that showed statistically significant overall effects.

Missing data were addressed as in the primary study (Jeppesen et al., 2025) using multiple imputations and incorporating variables associated with attrition at post-treatment into the statistical model. For each variable, 100 Markov Chain Monte Carlo imputations were performed.

The FaceYourFears trial was not powered for moderation analyses, underscoring the current study’s exploratory nature. Given the exploratory design, multiplicity adjustments were not applied (Bender & Lange, 2001).

#### Covariate analyses (variables associated with outcome irrespective of intervention)

In these analyses, associations between candidate demographic (age, gender, education, and occupation) and clinical (diagnosis, paranoia, social anxiety, depression, safety behavior, core beliefs, level of functioning, delusion severity, negative symptoms, anhedonia, avolition, cognitive biases, and interpersonal trauma) variables and post-treatment paranoia (GPTS) were examined. The regression coefficient (*b*-value) represents the relationship between the candidate baseline variable and post-treatment paranoia. Positive *b*-values indicate that a 1-point increase in the candidate baseline variable is associated with an increase in post-treatment GPTS, *suggesting* a poorer treatment response. Conversely, negative *b*-values indicate that a 1-point increase in the candidate baseline variable is associated with a decrease in post-treatment GPTS, *suggesting* a greater treatment response.

#### Moderator analyses (variables potentially modifying the effect of the intervention)

In these analyses, treatment group (VR-CBTp *or* CBTp) was the input variable, post-treatment paranoia (GPTS) was the output variable, and baseline demographic (age, gender, education, and occupation) and clinical (diagnosis, paranoia, social anxiety, depression, safety behavior, core beliefs, level of functioning, delusion severity, negative symptoms, anhedonia, avolition, cognitive biases, and interpersonal trauma) variables were candidate moderators. Moderators are factors that allow estimation of an effect for a specific intervention for a group of patients with specific factors (Van Hoorn et al., 2017, p. 2). The values of *b* represent the regression coefficients for the treatment group (VR-CBTp *or* CBT). CBTp served as the reference group; positive *b*-values indicate stronger associations in CBTp, while negative *b*-values indicate stronger associations in VR-CBTp.

Interaction analyses examined the relationship between treatment group allocation (randomization) and each moderator variable (randomization × moderator). For continuous variables, estimates of the outcome’s effect were conditioned at the 25th, 50th, and 75th percentile splits, representing ‘low’, ‘moderate’, and ‘high’ levels in the distribution of the candidate variables at baseline. These variables were analyzed as continuous moderators to assess linear associations across the full range of values. Separate analyses for each quartile were conducted, but results were only inspected in detail when interactions were significant.

## Results

In total, 254 participants were included in the analyses (Table 1), with no significant baseline demographic or clinical differences between the VR-CBTp and CBTp groups, as reported elsewhere (Jeppesen et al., 2025).

### Baseline variables associated with outcome

All outcomes for candidate baseline variables associated with the outcome are provided in Table 2a, with post hoc analyses in Table 2b. Below, the results of variables with statistically significant associations with post-treatment paranoia are summarized.

**Table 2a.** Results of covariate analyses (baseline variables associated with outcome irrespective of intervention).

Baseline candidate variables (continuous)	*B*	CI, Low	CI, High	*P*
Age	0.13	−0.01	0.27	0.08
Baseline paranoid ideation, R-GPTS	−0.23	−0.46	0.00	0.055
Baseline social anxiety, SIAS	0.07	−0.03	0.17	0.14
Baseline depressive symptoms, CDSS	0.04	−0.36	0.43	0.86
Baseline safety behavior, SBQ	0.07	0.01	0.13	**0.02**
Baseline positive self-beliefs, BCSS	0.16	−0.12	0.45	0.26
Baseline negative self-beliefs, BCSS	−0.02	−0.30	0.26	0.86
Baseline positive other beliefs, BCSS	−0.14	−0.49	0.22	0.45
Baseline negative other beliefs, BCSS	0.22	−0.08	0.52	0.14
Baseline level of functioning, PSP	−0.04	−0.15	0.08	0.52
Baseline delusion severity, SAPS	1.62	0.02	3.22	**0.047**
Baseline negative symptoms, BNSS	0.03	−0.11	0.17	0.69
Baseline anhedonia, BNSS	−0.09	−1.20	1.02	0.88
Baseline avolition, BNSS	1.36	0.03	2.70	**0.046**
Baseline cognitive bias, DACOBS	0.07	0.01	0.13	**0.01**
Baseline interpersonal trauma, TALE	−0.31	−0.91	0.30	0.32
*Baseline candidate variables (categorical)*				
Gender				
Female	1 (ref.)			
Male	−0.4	−3.17	2.44	0.80
Education				
Primary	1 (ref.)			0.88
Vocational	1.62	−3.24	6.48	0.51
Secondary/high school	0.26	−3.19 s	3.70	0.88
Post-secondary	−1.47	−5.89	2.95	0.51
Other	−0.41	−12.47	11.66	0.95
Diagnosis				
Schizophrenia (F20.0–9)	1 (ref.)			0.49
Schizotypy (F21)	−2	−5.84	1.88	0.31
Other (F22–9)	−2.1	−7.13	3.03	0.43

Significance level: *p* < 0.05 (highlighted). All confidence intervals (CI) set at 95%. Measures: BCSS: Brief Core Schema Scale; BNSS: Brief Negative Symptom Scale; CDSS: Calgary Depression Scale for Schizophrenia; DACOBS: Davos Assessment of Cognitive Biases Scale; R-GPTS: Revised Green Paranoid Thoughts Scale; PSP: Personal and Social Performance Scale; Safety Behavior Questionnaire; SAPS: Scale for the Assessment of Positive Symptoms; SIAS: Social Interaction Anxiety Scale; TALE: Trauma and Life Events Checklist.

**Table 2b.** Post hoc covariate analyses (baseline variables associated with outcome irrespective of intervention).

Baseline candidate variables	*B*	CI, Low	CI, High	*P*
SBQ, avoidance	0.13	0.00	0.27	0.06
SAPS, persecutory item	1.62	0.16	3.07	**0.03**
SAPS, self-referential item	0.31	−0.81	1.43	0.58
DACOBS, external attribution	0.15	−0.11	0.40	0.26
DACOBS, belief inflexibility	0.18	−0.09	0.45	0.18
DACOBS, jumping to conclusions	0.19	−0.08	0.46	0.17
DACOBS, safety behaviors	0.29	0.07	0.50	**0.01**
DACOBS, social cognitive problems	0.16	−0.11	0.43	0.25
DACOBS, subjective cognitive problems	0.02	−0.21	0.25	0.87
DACOBS, attention to threat	0.36	0.08	0.64	**0.01**

Significance level: *p* < 0.05 (highlighted). All confidence intervals (CI) set at 95%. Measures: DACOBS: Davos Assessment of Cognitive Biases Scale; SAPS: Scale for the Assessment of Positive Symptoms; SBQ: Safety Behavior Questionnaire.

Avolition, delusion severity, safety behavior, and cognitive biases were associated with outcome, with higher baseline levels being associated with higher post-treatment paranoia, indicating less treatment response. With associations in the same direction, post-hoc analyses showed that SAPS delusion subscale item persecutory delusions and DACOBS subscales attention to threat and safety behavior were associated with outcome.

### Moderation

#### Interactions

Results of interaction analyses for candidate moderators by treatment allocation are presented in Tables 3a and 3b.

**Table 3a.** Results of moderator analyses (baseline variables interacting with randomization), continuous variables.

	Interaction					< Median				≥ Median				< 25%ile				≥ 25%ile				≤ 75%ile				> 75%ile		
Baseline candidate moderator	Cont	*M*	25%	75%	*B*	CI, Lo	CI, Hi	*P*	*B*	CI, Lo	CI, Hi	*P*	*B*	CI, Lo	CI, Hi	*P*	*B*	CI, Lo	CI, Hi	*P*	*B*	CI, Lo	CI, Hi	*P*	*B*	CI, Lo	CI, Hi	*P*
Age	0.54	0.34	0.47	0.82																								
Baseline paranoid ideation, R-GPTS	0.48	0.55	0.44	0.32																								
Baseline social anxiety, SIAS	0.34	0.76	0.63	0.14																								
Baseline depressive symptoms, CDSS	0.43	0.29	0.45	0.56																								
Baseline safety behavior, SBQ	0.32	0.49	0.90	0.20																								
Baseline positive self-beliefs, BCSS	0.63	0.20	0.81	0.33																								
Baseline negative self-beliefs, BCSS	0.84	0.49	0.83	0.95																								
Baseline positive other beliefs, BCSS	0.45	0.24	0.64	0.56																								
Baseline negative other beliefs, BCSS	0.24	0.17	0.86	**0.04**	−1.14	−5.67	3.39	0.62	2.54	−1.08	6.17	0.17	1.24	−2.22	4.71	0.48	0.17	−4.66	5.01	0.94	2.60	−0.49	5.69	0.10	−5.02	−12.33	2.29	0.17
Baseline level of functioning, PSP	0.24	0.89	0.10	0.82																								
Baseline delusion severity, SAPS	**0.02**	0.12	**0.003**	0.12	6.01	−2.13	14.15	0.14	0.29	−2.70	3.28	0.85	4.16	0.71	7.60	**0.02**	−5.63	−10.51	−0.75	**0.02**	0.29	−2.70	3.28	0.85	6.01	−2.13	14.15	0.14
Baseline negative symptoms, BNSS	0.61	0.87	0.90	0.18																								
Baseline anhedonia, BNSS	0.56	0.66	0.48	0.84																								
Baseline avolition, BNSS	0.15	0.53	0.83	**0.01**	−0.23	−5.24	4.78	0.93	1.65	−1.72	5.02	0.33	1.55	−1.81	4.90	0.36	0.93	−4.61	6.47	0.74	2.73	−0.35	5.80	0.08	−7.05	−13.74	−0.37	**0.04**
Baseline cognitive bias, DACOBS	0.30	0.65	0.60	0.24																								
Baseline interpersonal trauma, TALE	0.62	0.94	0.85	0.54																								

Significance level: *p* < 0.05 (highlighted). All confidence intervals (CI) set at 95%. Abbreviations: Cont = continuous; DNC = ‘Does not compute’ (software unable to conduct analysis); Hi/H = high; Lo/L = low; *M* = median split; 25% = 25 percentile split; 75% = 75 percentile split. Measures: BCSS: Brief Core Schema Scale; BNSS: Brief Negative Symptom Scale; CDSS: Calgary Depression Scale for Schizophrenia; DACOBS: Davos Assessment of Cognitive Biases Scale; R-GPTS: Revised Green Paranoid Thoughts Scale; PSP: Personal and Social Performance Scale; Safety Behavior Questionnaire; SAPS: Scale for the Assessment of Positive Symptoms; SIAS: Social Interaction Anxiety Scale; TALE: Trauma and Life Events Checklist.

**Table 3b.** Results of moderator analyses (baseline variables interacting with randomization), categorical variables.

Baseline candidate moderator	Interaction		Predictor = 0 (no/not present)				Predictor = 1 (yes/present)										
	*P*	*B*	CI, Low	CI, High	*P*	*B*	CI, Low	CI, High	*P*								
Gender (male)	0.12																
			*Schizophrenia (F20.x)*				*Skizotypy (F21)*				*Other (F22–9)*						
	*P*	*B*	CI, Low	CI, High	*P*	*B*	CI, Low	CI, High	*P*	*B*	CI, Low	CI, High	*P*				
Diagnosis	0.07																
			*Primary*				*Vocational*				*Secondary*				*Other*		
	*P*	*B*	CI, Low	CI, High	*P*	*B*	CI, Low	CI, High	*P*	*B*	CI, Low	CI, High	*P*	*B*	*CI, Low*	CI, High	*P*
Level of education – participants	0.38																

Significance level: *p* < 0.05 (highlighted). All confidence intervals (CI) set at 95%.

**Table 3c.** Post hoc moderator analyses (baseline variables interacting with randomization), continuous variables.

	Interaction					< Median				≥ Median				< 25%ile				≥ 25%ile				≤ 75%ile				> 75%ile		
Baseline candidate moderator	Cont	*M*	25%	75%	*B*	CI, Lo	CI, Hi	*P*	*B*	CI, Lo	CI, Hi	*P*	*B*	CI, Lo	CI, Hi	*P*	*B*	CI, Lo	CI, Hi	*P*	*B*	CI, Lo	CI, Hi	*P*	*B*	CI, Lo	CI, Hi	*P*
SAPS, persecutory item	0.26	**0.04**	0.42	**0.04**	9.03	−2.51	20.57	0.12	0.32	−2.58	3.21	0.83	2.19	−1.62	6.01	0.26	−0.27	−4.21	3.68	0.89	0.32	−2.58	3.21	0.83	9.03	−2.51	20.57	0.12
SAPS, self-referential item	0.96	0.16	0.48	DNC																								

Significance level: *p* < 0.05 (highlighted). All confidence intervals (CI) set at 95%. Abbreviations: Cont = continuous; DNC = Does not compute’ (software unable to conduct analysis); Hi/H = high; Lo/L = low; *M* = Median split; 25% = 25 percentile split; 75% = 75 percentile split. Measures: SAPS: Scale for the Assessment of Positive Symptoms.

Interactions were observed for avolition, negative other beliefs, delusion severity, and post hoc persecutory delusions, indicating that these baseline variables were differentially associated with outcome when comparing VR-CBTp and CBTp.

#### Moderators of outcome following either CBTp or VR-CBT

All outcomes of candidate moderators stratified by percentiles are provided in Tables 3a and 3b, with post hoc analyses in Table 3c. Below, statistically significant interactions are summarized, some showing stronger associations in either VR-CBTp or CBTp.

Avolition significantly moderated the association between group and paranoia at treatment cessation at the 75th percentile. Avolition at the sample’s highest level was more strongly positively associated with outcomes in the VR-CBTp group than the CBTp group, indicating greater improvement following VR-CBTp for participants with high baseline avolition.

Delusion severity significantly moderated the association between group and paranoia at treatment cessation when analyzed at the 25th percentile. Delusions at the sample’s lowest level (<25th percentile) were more strongly positively associated with outcomes following CBTp compared to VR-CBTp, suggesting that participants with low delusion severity improved more in CBTp. However, delusions above the sample’s lowest level (≥25th percentile) were more strongly positively associated with outcomes in the VR-CBTp group, suggesting that participants with moderate-to-high levels improved more in VR-CBTp.

### Supplemental analyses

Supplemental analyses showed that SAPS global positive symptoms, BNSS asociality, alcohol intake, and parental education were associated with outcome (Supplementary Appendices B and C), and that SAPS global positive symptoms, BNSS asociality, DACOBS external attribution bias, and trustworthiness interacted with randomization (Supplementary Appendices D and E).

## Discussion

This exploratory study examined whether baseline demographic and clinical variables were associated with post-treatment paranoia and whether they interacted with treatment allocation following 10 sessions of VR-CBTp or standard CBTp in a sample diagnosed with SSD. Across treatments, baseline avolition, safety behaviors, cognitive biases, and delusion severity were associated with poorer outcomes. Separately, high baseline avolition was associated with better outcomes in VR-CBTp than CBTp, and, while low-to-moderate delusion severity was linked to better outcomes in VR-CBTp, lower delusion severity was linked to better response to CBTp. Post hoc analyses suggested that the severity of persecutory delusions was the primary contributor to this effect. Demographic (age, gender, and education) and other clinical variables (diagnosis, paranoia, social anxiety, depression, functioning, total negative symptoms, anhedonia, core beliefs, and interpersonal trauma) were unrelated to outcome.

### Baseline variables associated with outcome irrespective of treatment allocation

A novel finding was that higher baseline safety behavior was associated with *poorer* treatment outcomes across both CBTp and VR-CBTp but demonstrated no moderating effect. This contrasts previous findings suggesting it to be a moderator associated with *better* outcomes in VR-based interventions (Berkhof et al., 2024; Freeman et al., 2022). Compared with prior studies, the present study differed with respect to analytic strategy, number of sessions, measures of baseline paranoia, and administration of safety behavior subscales. Differences in primary symptom targets, such as agoraphobia versus paranoia, may also contribute. Safety behaviors related specifically to paranoia may constitute a distinct construct, and a dedicated self-report measure has recently been developed (Lambe et al., 2025). Given heterogeneity across studies, these findings should be interpreted cautiously. Further research is warranted to clarify how safety behaviors are associated with CBTp and VR-CBTp.

The negative effect of avolition mirrors prior findings linking higher baseline avolition to greater negative symptom severity at follow-up (Allott et al., 2011). Extending this to paranoia at treatment cessation, the present findings support the view that avolition represents a central domain within the negative symptom construct (Strauss et al., 2021). Taken together with the null findings for total negative symptoms, which contrast with earlier studies (Lincoln et al., 2014; Tarrier et al., 1993; Thomas et al., 2011), the results underscore the importance of assessing negative symptoms at the domain level (Galderisi et al., 2021), rather than relying solely on total scores or global ratings.

Consistent with prior findings (e.g. Brabban et al., 2009; Jenner et al., 2024), delusion severity was associated with outcome, with post hoc analyses suggesting the strength of perceived persecution to be involved. This is in line with cognitive models of persecutory delusions, which highlight their strong links with maintenance processes (e.g. Freeman, 2016). Future research could further examine how different components of persecutory delusion severity relate to outcome, with recent findings suggesting that conviction may be particularly prognostically informative (Jenner et al., 2024).

Cognitive biases were associated with outcome, consistent with their established role in the development and maintenance of delusions (De Rossi & Georgiades, 2022), but contrasting with previous findings suggesting that they do not constitute a barrier to improvement during CBTp (Lincoln et al., 2014). Post hoc analyses of DACOBS subscales indicated that ‘attention to threat’ and ‘safety behavior’ biases were particularly implicated. These subscales have previously shown strong correlations with clinician-rated safety behavior and self-reported paranoia (Pot-Kolder et al., 2018; Van der Gaag et al., 2013). Methodologically, this raises whether DACOBS provides incremental value when the GPTS and SBQ are already included, given that the GPTS is a preferred self-report measure of paranoia (Statham et al., 2019). Clinically and theoretically, these results highlight the importance of targeting specific threats to maintain cognitive processes in the treatment of paranoia (Moritz et al., 2025; Sauve et al., 2020), consistent with modular approaches aimed at strengthening a sense of safety as a counterbalance to heightened threat sensitivity (Freeman et al., 2021).

### Moderation of outcome following VR-CBTp or CBTp

The differential moderation by baseline avolition represents a novel contribution to CBTp research, as it suggests that VR-CBTp may mitigate the otherwise negative association between avolition and treatment outcome when compared directly with CBTp. This might reflect the greater in-session exposure time observed in VR-CBTp (Jeppesen et al., 2025), as well as its immersive, behavior-focused format, which can facilitate engagement in and accessibility to therapeutic tasks (Bell et al., 2024). In the FaceYourFears trial, VR-CBTp enabled real-time exposure and cognitive work during sessions, while CBTp relied more on between-session tasks such as homework and symptom monitoring (Jeppesen et al., 2022). Together with the cognitive restructuring demands typically required during CBTp sessions (e.g. Beck & Rector, 2005), these between-session requirements may pose barriers for individuals with motivational impairments, potentially limiting treatment outcomes (Kazantzis et al., 2016; Thai et al., 2024). This aligns with emerging evidence suggesting that immersive VR-interventions may help alleviate negative symptoms and the associated challenges (Novo et al., 2021). Given evidence linking therapeutic alliance with improvements in negative symptoms (Browne et al., 2021), and suggestions that VR may facilitate collaborative therapeutic experiences (e.g. Christensen et al., 2025), the attenuated negative impact of avolition in VR-CBTp relative to CBTp may partly reflect VR-specific affordances for strengthening alliance and engagement.

The differential moderation by baseline delusion severity also represents a novel finding, indicating a nuanced pattern of treatment responsiveness. This might reflect individuals with lower delusion severity having a stronger cognitive foundation, including greater cognitive flexibility, better insight, and lower conviction in their beliefs, which may facilitate engagement with the cognitive demands of CBTp (Brabban et al., 2009; Garety et al., 1997; Jenner et al., 2024; Naeem et al., 2008; Waltz, 2017). In contrast, individuals with more pronounced delusion symptoms may benefit more from the enhanced experiential learning offered by VR-CBTp, which can present safer, more immediate alternative experiences to challenge inflexible paranoid beliefs (Bell et al., 2024; Freeman et al., 2004). Thus, the present results hold promise for implementing precision medicine approaches in which either CBTp or VR-CBTp could be prescribed pending the initial level of paranoia severity.

### Nonsignificant treatment effect modification

Overall, most candidate baseline variables were unrelated to treatment outcome, aligning with meta-analytic evidence suggesting no consistent treatment effect modification of CBTp (Turner et al., 2020; Varese et al., 2025). However, the significant effects identified here suggest that the question remains open and highlight limitations in comparing CBTp protocols across studies. Differences in outcome measures, symptom targets, and conceptualization of symptoms may contribute to inconsistent findings. Furthermore, the integration of VR within CBTp has enabled examination of moderators less commonly assessed in traditional trials, such as safety behaviors (Berkhof et al., 2024), not explored in previous reviews or meta-analyses.

Notably, self-reported paranoia, which was a part of the inclusion criteria in the FaceYourFears trial (Jeppesen et al., 2025), was unrelated to outcome. This indicates that alternative cutoff thresholds of self-reported paranoia would most likely not have altered the conclusion that VR-CBTp was not superior to standard CBTp. These findings suggest that future VR-CBTp trials may consider alternatives to self-reported paranoia as eligibility criteria. Clinician-rated delusional severity may represent a more informative candidate, given its association with outcome, and may further enhance external validity by better aligning trial inclusion criteria with routine clinical assessment practices.

Given that the relatively weak moderation signals between VR-CBTp and CBTp provide little guidance for treatment selection in routine care, future research should also consider patient preference as a potential determinant of outcomes. Patient preference has been associated with improved treatment satisfaction, treatment completion, and clinical response (Bennett et al., 2025). Greater patient involvement in therapy processes, shared decision-making approaches, and allowing patients to select preferred treatment modalities may therefore enhance engagement and improve outcomes (Gerger et al., 2020; Stovell et al., 2016; Swift et al., 2018).

### Strengths and limitations

The study’s strengths include the clinical relevance, sample size, use of a broad range of candidate baseline variables, and data from the high-quality FaceYourFears trial, which enrolled 254 participants broadly representative of the psychiatric clinic population, enhancing generalizability.

Limitations should, however, be noted. First, the study was not powered to detect subgroup effects, as FaceYourFears employed a fixed sample size and was not designed for moderator analyses. No individual subgroup reached the required size for adequate statistical power, increasing the risk of type I and II errors. Moreover, interaction tests typically require larger sample sizes than those available. Additionally, the randomization has technically been compromised due to subgroup and interaction analyses, except when the subgroups were defined by a variable used for stratification during the original randomization procedure. Consequently, these exploratory subgroup analyses should be considered hypothesis-generating and require replication in adequately powered trials specifically designed to test subgroup effects. Furthermore, the statistical models used in the study could not differentiate between a lack of treatment response and clinical deterioration. Although baseline paranoia was controlled for and changes were interpreted as treatment effects, deterioration cannot be entirely excluded. However, given the large overall effect size in the primary trial (Jeppesen et al., 2025), substantial deterioration appears unlikely. All participants were included in the analyses, consistent with the intention-to-treat approach in the primary trial. While this preserves randomization benefits, minimizes bias, and enhances generalizability, it limits our ability to determine whether participants with certain moderating characteristics may have received suboptimal treatment or dropped out prematurely, potentially masking moderator effects. Moreover, the strength of the identified associations with the outcome seems modest. Although statistically significant, the clinical significance of identified associations between treatment outcome and baseline safety behavior, avolition, cognitive biases, and delusion severity appears modest. Finally, the analyses focused on baseline variables and treatment cessation outcome, preventing the assessment of in-session variables and long-term effects of candidate variables. Future studies should investigate in-session variables and whether baseline variables have sustained effects beyond the immediate post-treatment period.

## Conclusion

This exploratory study found that higher baseline avolition, safety behavior, delusion severity, and cognitive biases were associated with poorer paranoia outcomes across participants receiving 10 sessions of either CBTp or VR-CBTp. Moderator analyses revealed a more nuanced pattern: individuals with elevated avolition benefited more from VR-CBTp, and, while moderate-to-high delusion severity was linked to better outcomes in VR-CBTp, lower delusion severity was linked to better response to CBTp. The seemingly contradictory findings suggest that although higher avolition and delusion severity are generally linked to poorer outcomes overall, individuals with elevated levels may benefit relatively more from VR-CBTp than from CBTp in direct comparisons. Demographic and most clinical variables were unrelated to outcome, with significant associations being statistically or clinically modest. This tentatively suggests both therapies may be broadly applicable across diverse clinical profiles in individuals with paranoia. However, given the exploratory design, findings should be interpreted with caution and considered hypothesis-generating rather than conclusive.

## Supporting information

10.1017/S0033291726103870.sm001 Christensen et al. supplementary material Christensen et al. supplementary material

## Data Availability

Data from the FaceYourFears trial will, after initial publications, be made publicly available through the Danish National Archives.

## Supplementary material

The supplementary material for this article can be found at http://doi.org/10.1017/S0033291726103870.

## Clinical trial registration

The FaceYourFears trial was registered with ClinicalTrials.gov identifier NCT04902066.
